# Surgical management versus non-surgical management of rib fractures in chest trauma:a systematic review and meta-analysis

**DOI:** 10.1186/s13019-019-0865-3

**Published:** 2019-02-27

**Authors:** Xin Liu, Kai Xiong

**Affiliations:** 1 The Second Hospital of TianJin Medical University, Ping Jiang Road No.23, Hexi District, Tianjin, 300211 China; 2 Tianjin Medical University General Hospital, An Shan Road No.154, Heping District, Tianjin, 300192 China

**Keywords:** Rib fractures, Surgical management, Non-surgical management, Complication, Meta-analysis

## Abstract

**Objective:**

Rib fractures are common injuries sustained by patients who experience high-impact chest trauma, and they result in severe respiratory compromise because of the altered mechanics of respiration. Several studies have shown that the ventilation requirements and incidence of pulmonary complications may be decreased with operative intervention. The purpose of this study was to evaluate the effect of surgical fixation treatments for rib fractures through systematic review and meta-analysis.

**Methods:**

A literature search was performed in the PubMed, EMBASE, Web of Science and Cochrane Library databases for information from February 1958 to April 2018. Studies comparing the benefits of surgical management with that of non-surgical management of rib fractures were included. Statistical heterogeneity was evaluated by the X^2^ test with the significance set to *P* < 0.10 or I^2^ > 50%.

**Results:**

Fourteen studies consisting of 839 patients were included (407 patients in the surgical management group; 432 patients in the non-surgical management group). The results showed that the surgical management group experienced a significant decrease in hospitalization time, intensive care time, mechanical ventilation time, mortality rate, pulmonary infection rate and tracheotomy rate compared with the non-surgical management group. However, the surgical management group incurred extra costs, and there was no significant difference in the duration of antibiotic use between the two groups.

**Conclusions:**

Compared with non-surgical management, surgical management methods are of great value in the treatment of rib fractures despite the added expense.

## Key question

Compare and analyse the recovery rate and rate of complications of rib fractures managed with surgical and non-surgical treatments.

## Key findings

Surgical management will provide more benefits for patients with rib fractures.

## Take home message

Surgical management is more effective than non-operative management for rib fractures.

## Introduction

Rib fractures are among the most common traumatic injuries and are found in 20% of all patients who suffer thoracic trauma [1]. Rib fractures are responsible for significant loss of work days and can affect patients for several months after the injury. The incidence of rib fractures in closed thoracic trauma is as high as 85%. Common causes of injury include road traffic accidents, high falls, indirect crush forces, and direct violence. Rib fractures caused by these injuries are clinically divided into single or multiple rib fractures [2]. One special type of injury is flail chest (FC), which is defined as the fracture of three or more sequential ribs at multiple sites and results in paradoxical chest wall movement, altered respiratory mechanics, and, frequently, respiratory failure. The treatments for rib fractures are mainly divided into categories of conservative treatment and surgical treatment. Traditional conservative treatment methods include rib bone traction, pressure dressing and ventilator-assisted respiration; there are also many methods for surgical treatment [3]. In this context, there is much interest in which method is of greater value in the treatment of rib fractures.

## Materials and methods

### Literature and search strategy

Two different investigators independently searched the following electronic databases for information from February 1958 to April 2018: PubMed, EMBASE, Web of Science and the Cochrane Library. The following related terms were searched: Rib fractures; Surgical procedures, Operative; Conservative treatment; Flail chest; and Meta-analysis. A search strategy was constructed by combining the above terms with “AND” or “OR”. No restrictions were imposed on the language of the studies. We also screened the references lists of the retrieved articles so that relevant studies were not missed.

### Study selection criteria

Two different reviewers independently assessed the retrieved articles to determine whether the study met the inclusion criteria. In case of disagreements, a third reviewer was involved in the discussion until a consensus was reached. The inclusion criteria were as follows: (I) randomized controlled trials comparing surgical management with non-surgical management in adults with rib fractures; and (II) studies with patients who had no other concomitant diseases, such as respiratory dysfunction, liver or kidney dysfunction, etc. The exclusion criteria included the following: (I) case-control studies, animal studies, cadaver studies, single case reports, comments, letters, editorials, protocols, guidelines, publications based on surgical registries, and review papers; and (II) studies with patients who had the following characteristics: (i) coagulation disorders, instability of vital signs, infections or any surgical contraindications; (ii) thoracocyllosis; (iii) previous or current complicated severe pulmonary disease (such as emphysema, respiratory failure, etc.), circulatory system disease (such as myocardial infarction, heart failure, etc.), liver or kidney insufficiency, and tumours.

### Data extraction and quality assessment

Two different investigators independently performed the data extraction and methodological quality assessment. Data extracted from the included studies consisted of authors, publication date, study design, number of patients, surgical approach, follow-up duration and outcome data in both the surgical management group and the non-surgical management group. The outcome measures included duration of antibiotic use, incurred expense, hospitalization time, intensive care time, mechanical ventilation time, mortality rate, pulmonary infection rate and tracheotomy rate. The methodological quality of the study was evaluated in six domains, including sequence generation, allocation concealment, participant blinding, assessor blinding, incomplete data, selective reporting and other bias. Each included study could be considered as unclear risk, low risk or high risk of bias in each domain based on the Cochrane Handbook 5.1.0.

### Studies and patients

**Fig. 1:**
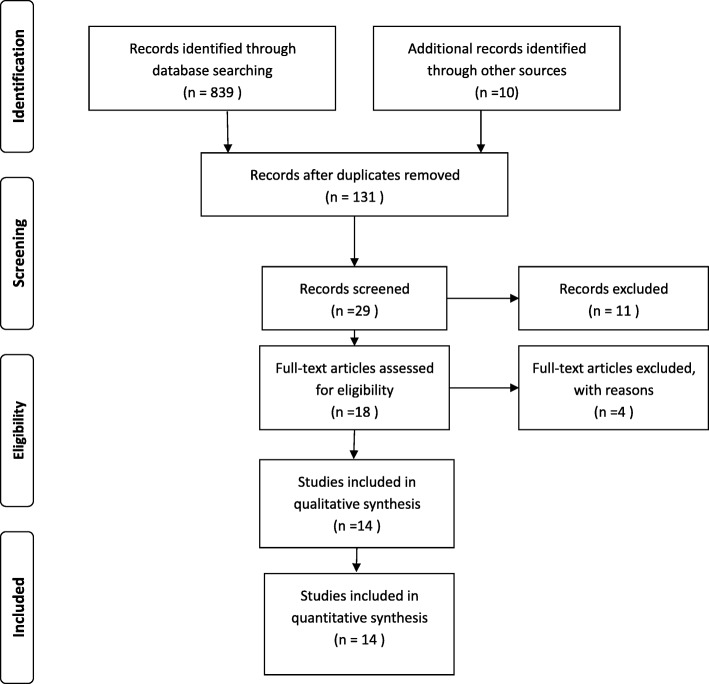
Flow chart illustrating the literature search process (surgical management and non-surgical management groups)

**Fig. 2:**
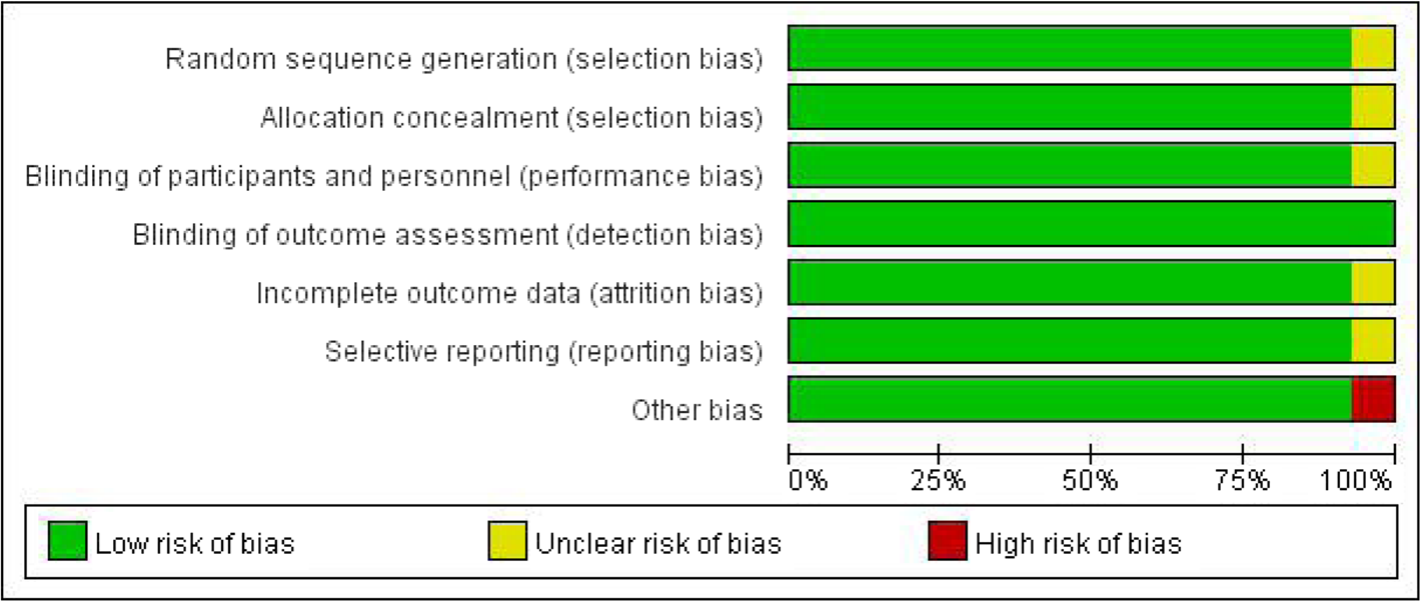
Risk of bias graph

**Fig. 3:**
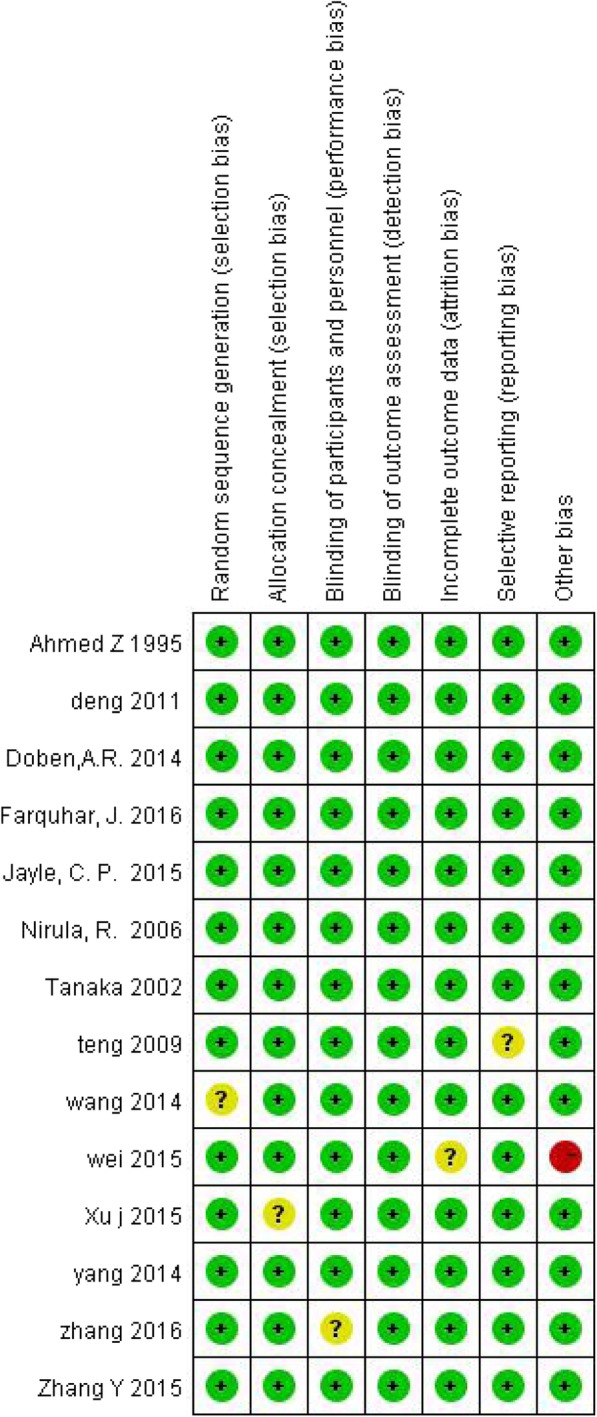
Risk of bias summary ( indicates a low risk of bias,  indicates a high risk of bias,  indicates an unclear or unknown risk of bias)

The retrieval strategy is displayed in Fig. 1. In total, 839 titles and abstracts were preliminarily reviewed, and the potentially eligible citations were searched online. Of these, 14 studies satisfied the eligibility criteria (407 surgical management group patients and 432 non-surgical management group patients). No other apparent biases were found among the included studies. Figures 2 and 3 show the summary for risks of bias. The reviewers independently applied the criteria described above and below to the full text of these articles to select the articles included in this review.

### Statistical analysis

Statistical analyses were performed using the procedures in the Review Manager Software 5.3. For dichotomous outcomes, the odds ratio (OR) with 95% confidence interval (CI) was calculated to estimate the pooled average difference between the surgical management and non-surgical management groups; weighted mean difference (WMD) with 95% CI were calculated for continuous outcomes. Statistical heterogeneity was quantitatively evaluated by the X^2^ test with the significance set at *P* < 0.10 or I^2^ > 50%. The data are presented in the form of forest plots.

## Results

Fourteen papers included analyses of the number of patients, patient sex, number of fractured ribs and study type. A detailed summary of the characteristics and results of the included studies is listed in Table 1. Table 2 shows the results of the indicators by the meta-analysis.

**Table 1:** Summary of the characteristics and results of the included studies

References	Patients (rib fractures)		Sex (male:female)		No of Fracture ribs	Study type
	SM	NSM	SM	NSM		
Ahmed.Z [1]	26	38	23:3	18:1	5 ~ 8.	Co
Deng [2]	16	18	NR	NR	> 3	Co
Doben.A.R [3]	10	11	9:1	7:4	3 in ≥2 places	Co
Farquhar.J [4].	19	36	15:4	25:11	> 3	Co
Jayle.C.P [5]	10	10	4:1	4:1	3 in ≥2 places	Co
Nirula.R [6].	30	30	NR	NR	NR	Co
Tanka [7]	18	19	2:1	14:5	> 6	RCT
Teng [8]	32	28	NR	NR	≥4	Co
Wang [9]	69	72	41:28	43:29	≥2	RCT
Wei [10]	50	54	30:20	37:17	≥3	Co
Xu.J [11]	17	15	12:5	12:3	≥4	Co
Yang [12]	30	30	20:10	21:9	≥3	Co
Zhang.Z.T [13]	56	56	37:19	31:25	2~6.	Co
Zhang Y [14]	24	15	19:5	14:1	7~16	Co

SM surgical management group that patients who are managed operatively,

NSM non-surgical management group that patients who are managed nonoperatively

NR not reported, RCT random control trail, Co cohort study

**Table 2:** Results of the meta analysis

Outcomes	Studies(n)	Events(n)	X^2^ (*P* value)	I^2^	Odds ratio (95% CI)
mortality	3	158	0.32	13	0.28 [0.08,0.92]
LHOF	7	471	0.19	32	−7.40[−8.51,-.6.28]
LIOF	6	209	0.17	35	−2.28[−3.26,-1.31]
DOMV	4	149	0.31	17	−4.68[−5.62,-3.75]
antibiotic use	2	71	0.49	0	−3.69[−7.63,0.26]
pulmonary infection	8	570	0.12	38	0.25 [0.16,0.39]
tracheotomy	3	133	0.39	0	0.14 [0.06,0.36]
incurred expense	3	198	0.27	0	0.27 [0.23,0.31]

*LHOF:the length of hospital stay; LIOF:the length of ICU stay; DOMV:duration of mechanical ventilation

**Fig. 4:**
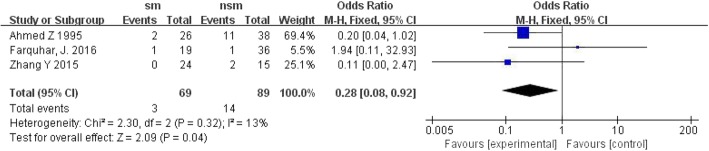
Forest plot of comparison: mortality rate(%). (*sm*:surgical management, *CI* confidence interval, *df* degrees of freedom)

Three trials reported mortality. The pooled results showed that the surgical management group had a lower mortality rate than the non-surgical management group, with OR = 0.28; 95% CI, 0.08 to 0.92; *P* < 0.1 and without significant heterogeneity (I^2^ = 13%, *P* = 0.04) (Fig. 4).

**Fig. 5:**
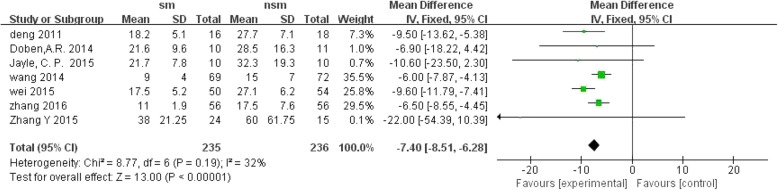
Forest plot comparison: the length of hospital stay(d). (*sm* surgical management, *CI* confidence interval, *df* degrees of freedom)

**Fig. 6:**
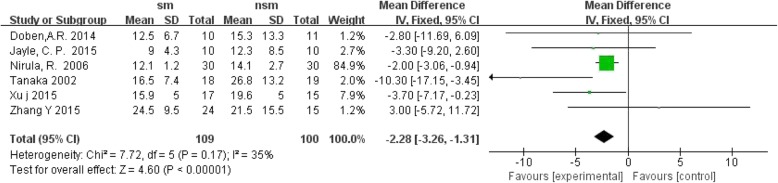
Forest plot comparison: the length of ICU stay(d). (*sm* surgical management, *CI* confidence interval, *df* degrees of freedom)

Seven trials reported the length of hospital stay. The results revealed that the surgical management group was associated with a greater decrease in the length of hospital stay, with OR = − 7.40; 95% CI, − 8.51 to − 6.28; *P* < 0.1 and without significant heterogeneity (I^2^ = 32%, *P* < 0.00001); the surgical management group was also associated with a shorter intensive care time, with OR = − 2.28; 95% CI, − 3.26 to − 1.31; *P* < 0.1 and without significant heterogeneity (I^2^ = 35%, *P* < 0.00001) (Figs. 5 and 6).

**Fig. 7:**
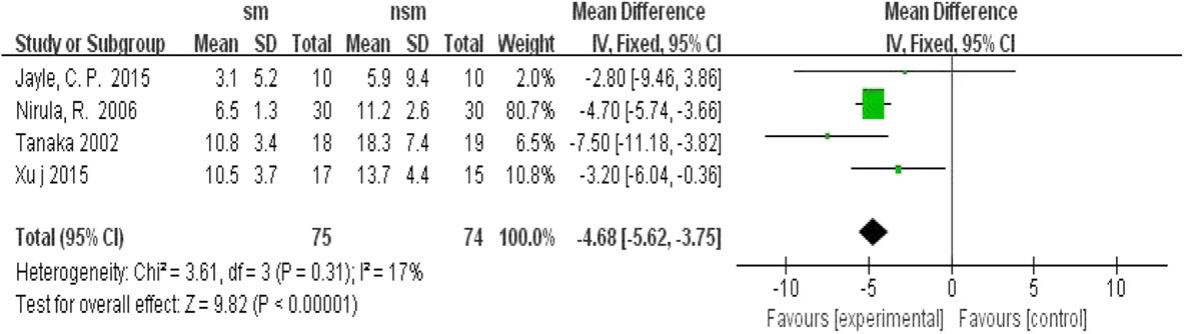
Forest plot comparison: duration of mechanical ventilation(h). (*sm* surgical management, *CI* confidence interval, *df* degrees of freedom)

Four trials reported the duration of mechanical ventilation. The results showed that the surgical management group had shorter DOMV, with OR = − 4.68; 95% CI, − 5.62 to − 3.75; *P* < 0.1, without significant heterogeneity (I^2^ = 17%, *P* < 0.00001) (Fig. 7).

**Fig. 8:**
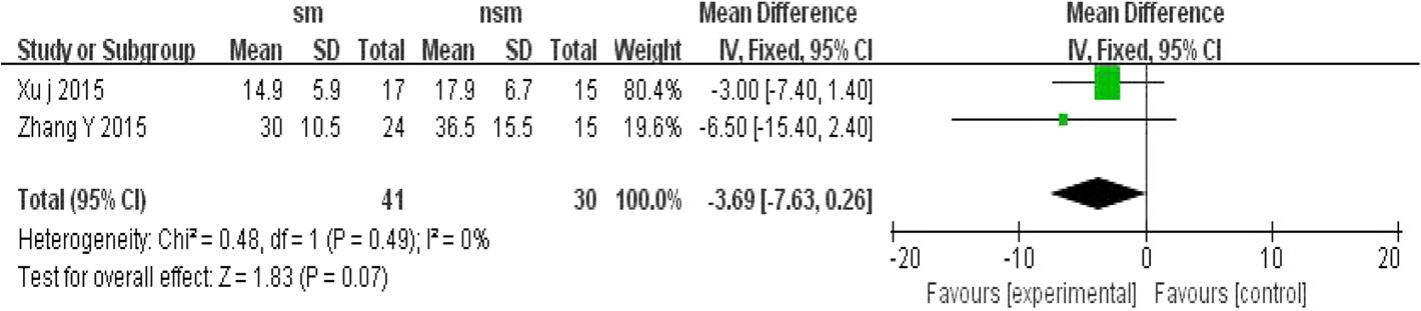
Forest plot comparison: duration of antibiotic use (d). (*sm* surgical management, *CI* confidence interval, *df* degrees of freedom)

Two studies with patients both reported that the duration of antibiotic use was not significantly different between the non-surgical management therapy group and the surgical management group, with OR = − 3.69; 95% CI, − 7.63 to 0.26; *P* < 0.1 and without significant heterogeneity (I^2^ = 0%, *P* = 0.07) (Fig. 8).

**Fig. 9:**
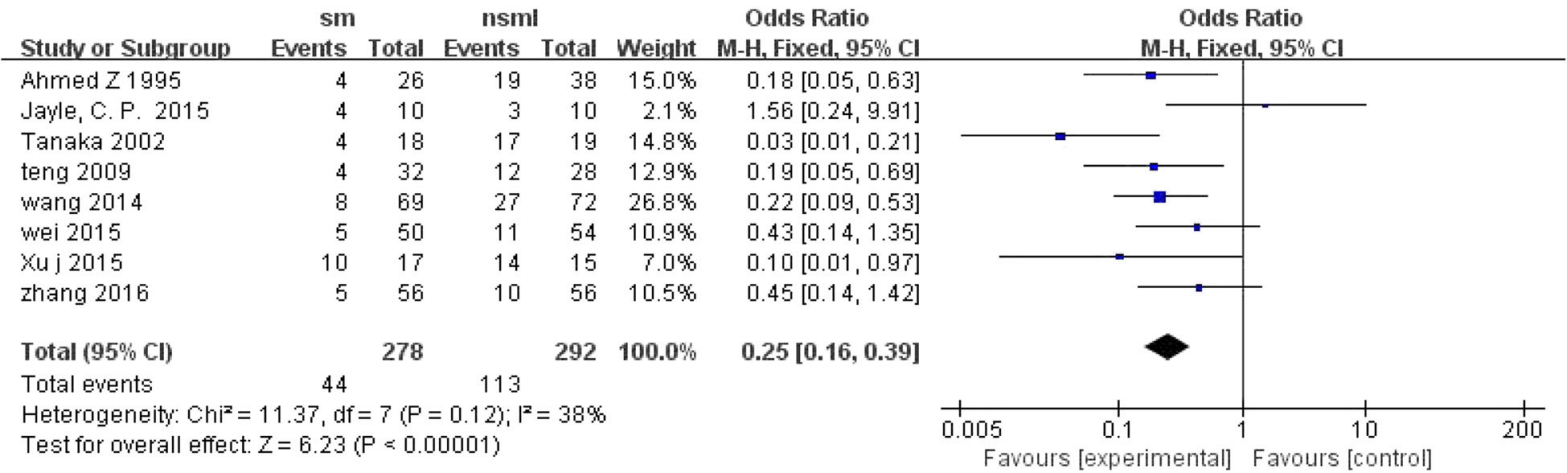
Forest plot comparison: pulmonary infection rate(%). (*sm* surgical management, *CI* confidence interval, *df* degrees of freedom)

The non-surgical management group had an even higher risk of pulmonary infection than the surgical management group, with OR = 0.25; 95% CI, 0.16 to 0.39; *P* < 0.1 and without significant heterogeneity (I^2^ = 38%, *P* < 0.00001) (Fig. 9).

**Fig. 10:**
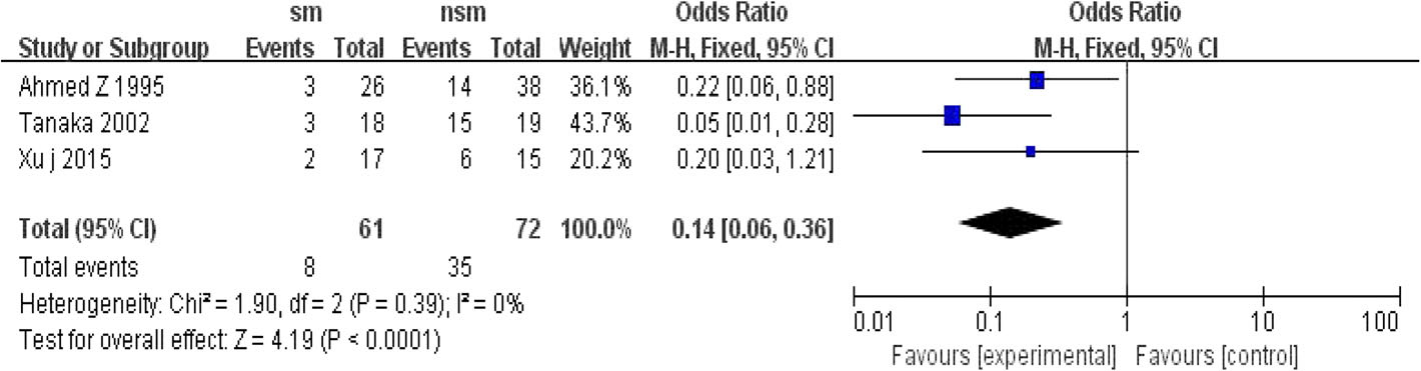
Forest plot comparison: tracheotomy occurrence rate(%). (*sm* surgical management, *CI* confidence interval, *df* degrees of freedom)

Four studies reported tracheotomy occurrences in the surgical management group and non-surgical management group. The incidence of tracheotomy was more frequent in the non-surgical management group than in the surgical management group, with OR = 0.14; 95% CI, 0.06 to 0.36; *P* < 0.1 and without significant heterogeneity (I^2^ = 0%, *P* < 0.0001) (Fig. 10).

**Fig. 11:**
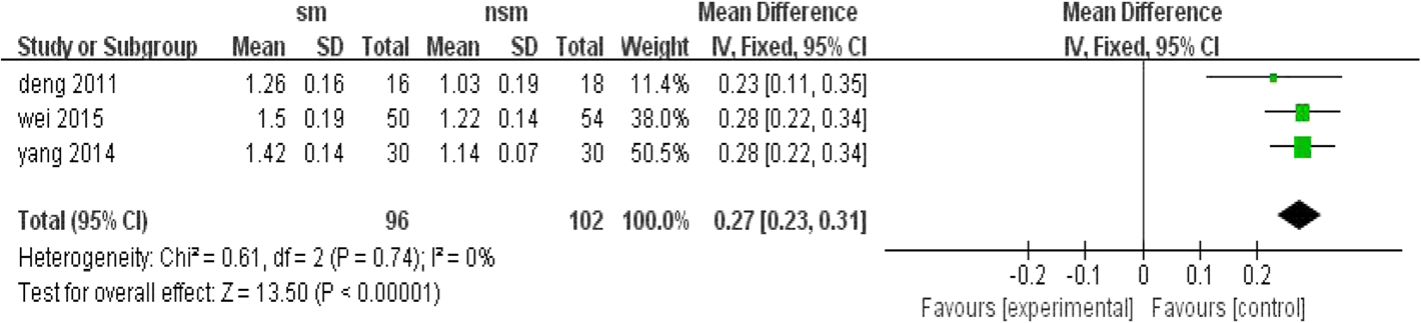
Forest plot comparison: incurred expense. (*sm* surgical management, *CI* confidence interval, *df* degrees of freedom)

Three studies reported the incurred expense and showed significant differences between the surgical management group and the non-surgical management group, with OR = 0.27; 95% CI, 0.23 to 0.31; *P* < 0.1 and without significant heterogeneity (I^2^ = 0%, *P* < 0.00001) (Fig. 11). Surgical intervention requires more expense.

## Discussion

Severe thoracic trauma is a common cause of chest injury. Patients with severe thoracic trauma combined with rib fractures who fail to receive effective treatment in time may develop complications such as a haemothorax and pneumothorax, which may seriously affect their prognosis [2].

In the past, conservative treatments for severe thoracic trauma with rib fractures was common, but the therapeutic effect was not satisfactory [4]. For patients treated non-surgically, although mechanical ventilation and external chest fixation partially relieved the pathophysiological changes caused by abnormal breathing and pulmonary contusions, these methods could not completely eliminate their abnormal breathing so mild activity could lead to severe pain; patients treated non-surgically need more potent analgesic drugs and cannot cope with coughing to expel the easily formed airway secretions, which can cause hypoxemia, severe pulmonary infections, atelectasis and other lung complications [5]. Meanwhile, the chest wall still has varying degrees of softening and collapse, and misplaced rib fractures can lead to thoracic deformities that affect appearance or even result in damage to the intercostal blood vessels and nerves [6].

In recent years, the method of open reduction and internal fixation for severe thoracic trauma combined with rib fractures has been widely used [3]. Studies have shown that 72 h after operation of a rib fracture, the pain caused by the friction between the broken ends of the fractures is reduced, the effect of mediastinal oscillate on the cardiovascular circulation is eliminated, the patient’s respiratory movement changes from shallow to normal, and the haemodynamics are significantly improved. Meanwhile, during general anaesthesia, the lung tissue showed good swelling, lung ventilation was improved, and management of the respiratory tract can effectively remove respiratory secretions in the acute phase [7]. Therefore, in some patients with mild pulmonary contusions, extubation was performed immediately following recovery from anaesthesia without the need for mechanical support. Surgically treated patients did not need external fixation and were not restricted in respiratory activity. Surgically treated patients could cough, turn over independently and get out of bed earlier than non-surgically treated patients. These activities are conducive to maintaining airway patency and reducing lung complications. As a result, the duration and use of mechanical ventilation in the surgical management group were significantly reduced, and there were clear advantages in pain control [8]. These reported results are consistent with the research results in this paper.

This meta-analysis presents results from 14 studies comparing surgical approaches to non-surgical approaches for the treatment of rib fractures [6–19]. We conclude that surgical intervention decreased the duration of hospitalization time, intensive care time, and mechanical ventilation time, lowered the odds for needing a tracheotomy and contracting a pulmonary infection, and reduced the mortality rate, as compared to non-surgical management.

In addition, many studies have shown the advantages of using surgical treatment for rib fractures. For example, Tanaka A. et al. [20] reported that through open reduction and internal fixation of rib fractures, the fractured end can reach anatomical reduction. Abnormal breathing disappears immediately after surgery, and the thorax restores its original shape and function, thereby greatly reducing the rate of tracheotomy. Moreover, surgical management can reduce the compression and stimulation of fractured intercostal nerves and effectively relieve respiratory pain so the airway secretions can be more easily coughed up; these factors will promote self-discharge, improve respiratory function, reduce the incidence of pulmonary complications, shorten the hospital stay, and improve the patient’s quality of life [21]. Treating multiple rib fractures with open reduction and internal fixation has been shown to be a more scientific and rational treatment method, especially for patients with serious fracture dislocations [22].

This study also has some limitations. It is difficult to design high-quality studies on the effect of surgical management on the outcomes of rib fractures because the participants could not be randomly assigned to exposure groups, and blinding is only partially possible. We chose to include all comparative studies in this systematic review since that represented the best evidence available at present. The differentiation between retrospective and prospective trials can be difficult because many authors present a study with prospective data collection and retrospective analysis of the data as being prospective in design. Scoring of the methodology, however, showed that the studies included in this review were comparable and that pooling the studies was therefore justifiable.

## Conclusion

This is a systematic study to report the effect of surgical treatment and non-surgical treatment of multiple rib fractures. The patients who had surgical treatment showed a greater decrease in mortality rate, hospitalization time, intensive care time, mechanical ventilation time, tracheotomy rate and pulmonary infection rate than patients who had non-surgical treatment, although surgical treatment costs more. Surgeons and patients can choose the best treatment method according to this conclusion to achieve more humane and effective treatment. More high-quality multi-centre prospective RCT (random control trial) s with good designs, a large number of participants and long-term follow-ups are necessary to confirm these conclusions in the future.

## Abbreviations

CI: Confidence interval
Co: Cohort study
df: Degrees of freedom
DOMV: Duration of mechanical ventilation
FC: Flail chest
LHOF: Length of hospital stay
LIOF: Length of ICU stay
NR: Not reported
NSM/nsm: Non-surgical management group, with patients who are managed non-operatively
OR: Odds ratio
RCT: Random control trial
SM/sm: Surgical management group, with patients who are managed operatively
WMD: Weighted mean difference
